# Transmembrane thioredoxin‐related protein TMX1 is reversibly oxidized in response to protein accumulation in the endoplasmic reticulum

**DOI:** 10.1002/2211-5463.12319

**Published:** 2017-10-03

**Authors:** Yoshiyuki Matsuo, Kiichi Hirota

**Affiliations:** ^1^ Department of Human Stress Response Science Institute of Biomedical Science Kansai Medical University Japan

**Keywords:** disulfide bond, endoplasmic reticulum, oxidoreductase, protein folding, redox

## Abstract

Numerous secretory and membrane proteins undergo post‐translational modifications in the endoplasmic reticulum (ER), and the formation of disulfide bonds is a modification that is critical for proper protein folding. The mammalian ER contains a large family of oxidoreductases that are considered to catalyze thiol/disulfide exchange and ensure the maintenance of a redox environment within the ER. Disruption of ER homeostasis causes an accumulation of misfolded and unfolded proteins, a condition termed ER stress. Despite advances in our understanding of the ER stress response and its downstream signaling pathway, it remains unclear how ER redox balance is controlled and restored in the stressed ER. In this study, we determined that brefeldin A (BFA)‐induced protein accumulation in the ER triggers reversible oxidation of transmembrane thioredoxin‐related protein 1 (TMX1). Conversion of TMX1 to the oxidized state preceded the induction of immunoglobulin‐binding protein, a downstream marker of ER stress. Oxidized TMX1 reverted to the basal reduced state after BFA removal, and our results suggest that glutathione is involved in maintaining TMX1 in the reduced form. These findings provide evidence for a redox imbalance caused by protein overload, and demonstrate the existence of a pathway that helps restore ER homeostasis during poststress recovery.

**Abbreviations**
AMS: 4‐acetamido‐4ʹ‐maleimidylstilbene‐2,2ʹ‐disulfonic acid
BFA: brefeldin A
BiP: immunoglobulin‐binding protein
BSO: l‐buthionine sulfoximine
DIA: diamide
eIF2: eukaryotic translation initiation factor 2
ER: endoplasmic reticulum
GSH: glutathione
MD: menadione
NAC: *N*‐acetyl‐l‐cysteine
NEM: *N*‐ethylmaleimide
PDI: protein disulfide isomerase
RFP: red fluorescent protein
ROS: reactive oxygen species
TCEP: tris(2‐carboxyethyl)phosphine hydrochloride
TG: thapsigargin
TM: tunicamycin
TMX: transmembrane thioredoxin‐related protein
Tub: α‐tubulin
UPR: unfolded protein response

In eukaryotic cells, the endoplasmic reticulum (ER) is the site of protein synthesis in the secretory pathway 1, 2. Proteins that are transported through the ER undergo various post‐translational modifications, including glycosylation and disulfide bond formation, which are critical for protein maturation and assembly 3. Environmental or pathological factors that perturb ER functions cause an accumulation of unfolded and misfolded proteins in the ER; this condition, named ER stress, activates an intracellular signaling pathway referred to as the unfolded protein response (UPR) 4, 5. The UPR induces the transcriptional activation of downstream effector molecules and thereby reduces the stress and restores ER homeostasis for adaptation and cell survival. However, prolonged activation of the UPR pathway could lead to apoptotic cell death, which has been implicated in the pathogenesis of diverse human chronic diseases 6.

Disulfide bonds are introduced into proteins through the oxidation of a pair of cysteine residues. The formation of protein disulfides is essential for proper protein folding and contributes to protein stability 7. Moreover, disulfide modification is crucial for the adaptive response against protein misfolding. Non‐native disulfide bonds are either reshuffled for regeneration or cleaved for subsequent proteasomal degradation 8, 9. The mammalian ER harbors a large family of oxidoreductases that contain a characteristic functional domain related to thioredoxin 10; these oxidoreductases are considered to be responsible for thiol/disulfide exchange, which enables the formation or cleavage of protein disulfide bonds. The oxidoreductase activity of these enzymes depends on the pair of cysteine residues present within the active‐site motif CXXC, which can shuttle between reduced and oxidized states. The redox property of the active site determines the catalytic activity of a given oxidoreductase, and the redox status of ER oxidoreductases is specifically manipulated by either enzymatically catalyzed pathways or nonenzymatic reactions 11.

Most ER oxidoreductases are retained within the ER, and previous studies have investigated the redox regulation of these enzymes localized within the luminal compartment of the ER. However, little is known regarding the mechanisms that regulate membrane‐anchored oxidoreductases of the transmembrane thioredoxin‐related protein (TMX) subfamily. TMX1 is a type I transmembrane protein that contains an N‐terminal signal sequence, a single thioredoxin‐like domain that features a catalytic CPAC active‐site motif, a transmembrane domain, and a C‐terminal cytosolic tail 12, 13. TMX1 is localized to the ER membrane and preferentially interacts with transmembrane polypeptides 14, 15. Recent studies revealed the recruitment of palmitoylated TMX1 to the mitochondria‐associated membrane, where it can function to regulate the calcium flux between the ER and mitochondria 16.

To extend our knowledge of how the activities of ER oxidoreductases are controlled, we focused on TMX1, which has been shown to exist predominantly in the reduced form at steady state 14. Here, we used TMX1 as a model that allowed us to monitor the ER redox environment. We examined how ER stress affects the redox status of TMX1, and we analyzed the process of TMX1 recovery from the oxidation caused by protein accumulation in the ER.

## Materials and methods

### Cell culture

Human lung adenocarcinoma cell line A549 cells and hepatocellular carcinoma cell line HepG2 cells were obtained from Health Science Research Resources Bank (Osaka, Japan). A549 and HepG2 cells were maintained in Dulbecco's modified Eagle's medium supplemented with 10% fetal bovine serum. Plasmids encoding myc‐tagged TMX1 (pEF6‐TMX1‐myc 14) were transfected with Lipofectamine 3000 Reagent (Thermo Fischer Scientific, Waltham, MA, USA) for transient expression.

### Reagents

The following stock solutions were prepared: 2 mg·mL^−1^ brefeldin A (BFA; Nacalai Tesque, Kyoto, Japan, 05325‐81) in methanol; 5 mm thapsigargin (Wako, Osaka, Japan, 209‐17281) in DMSO; 10 mg·mL^−1^ tunicamycin (Wako, 202‐08241) in DMSO; 1 m DTT (Wako) in water; 400 mm diamide (Sigma, St Louis, MO, USA, D3648) in PBS; 100 mm 4‐acetamido‐4ʹ‐maleimidylstilbene‐2,2ʹ‐disulfonic acid (AMS; Thermo Fischer Scientific, A485) in water; 10 mm menadione (Nacalai Tesque, 36405‐71) in DMSO; 100 mm l‐buthionine sulfoximine (BSO; Wako, 021‐14121) in water; and 500 mm *N*‐acetyl‐l‐cysteine (NAC; Sigma, A9165) in 1 m HEPES. *N*‐Ethylmaleimide (NEM) was obtained from Wako (058‐02061), and Bond‐Breaker TCEP solution from Thermo Fischer Scientific (77720).

### Antibodies

The following antibodies were used: anti‐TMX1 (SIGMA, HPA003085), anti‐BiP (BD Biosciences, San Jose, CA, USA, 610978), anti‐α‐tubulin (Wako, 017‐25031), anti‐PDI (Enzo Life Sciences, Farmingdale, NY, USA, SPA‐890), anti‐P5 (Thermo Fischer Scientific, PA3‐008), anti‐phospho‐eIF2α (Cell Signaling Technology, Danvers, MA, USA, 9721), and anti‐eIF2α (Cell Signaling Technology, 9722).

### Determination of redox state

TMX1 is a single‐pass transmembrane protein and is efficiently extracted with nonionic detergents 13. The redox state of TMX1 was determined as described previously 14. Briefly, cells were incubated with ice‐cold PBS containing 20 mm NEM to alkylate free thiols, and lysed in 50 mm HEPES, 150 mm NaCl, 20 mm NEM, and 0.5% NP‐40. Insoluble materials were precipitated by centrifuging the samples at 20 000 ***g*** for 5 min, and the resulting supernatants were used in immunoblotting analyses performed under nonreducing conditions. To assess the redox state of PDI and P5, alternative methods were used with a few modifications 17, 18. Cell lysates were prepared as described above, and SDS (1%) was added to denature proteins. Excess NEM was removed from the lysates by using a Zeba Spin Desalting Column (7K MWCO; Thermo Fischer Scientific) equilibrated with 50 mm HEPES, and 150 mm NaCl, and then, SDS (1%) and TCEP (10 mm) were added to the eluates and the samples were incubated at 25 °C for 15 min to reduce existing disulfide bonds. The samples were subject to a second alkylation with 15 mm AMS at 25 °C for 30 min and then analyzed by immunoblotting.

### Immunoblotting analysis

Samples were separated using SDS/PAGE and transferred to Immobilon‐P (Merck Millipore, Darmstadt, Germany) polyvinylidene fluoride membranes, which were blocked in Blocking One reagent (Nacalai Tesque). Blots were incubated with various antibodies, and chemiluminescent detection was performed using ECL Prime reagent (GE Healthcare, Little Chalfont, UK). Band intensities were quantified through densitometric analysis performed using image studio lite software (LI‐COR Biosciences, Lincoln, NE, USA).

### Fluorescence microscopy

A549 cells were transduced with CellLight Golgi‐RFP (Thermo Fischer Scientific, C10593) at a concentration of 40 particles per cell and cultured for 24 h. Subsequently, the cells were treated with vehicle or BFA (0.1 μg·mL^−1^) for 6 h, and then, nuclei were stained with PureBlu Hoechst 33342 (Bio‐Rad, Hercules, CA, USA, 135‐1304). After fixation in 4% paraformaldehyde, fluorescence images were acquired using a BZ‐9000 fluorescence microscope (KEYENCE, Osaka, Japan).

### Measurement of ROS production

A549 cells were treated with vehicle, BFA (0.1 μg·mL^−1^), or menadione (25 μm) for various periods, and the H_2_O_2_ level was measured by using the reactive oxygen species (ROS)‐Glo H_2_O_2_ Assay kit (Promega, Fitchburg, WI, USA) as per the manufacturer's instructions. Luminescence was recorded using an EnSpire Multimode Plate Reader (Perkin Elmer, Waltham, MA, USA). Each sample was assayed in triplicate, and the experiments were repeated twice.

### Glutathione assay

Cellular glutathione levels were quantified by using the glutathione (GSH)‐Glo Glutathione Assay kit (Promega) according to the manufacturer's protocol. Luminescence was recorded using the EnSpire Multimode Plate Reader. Each sample was assayed in triplicate, and the experiments were repeated thrice.

### Statistical analysis

Data are expressed here as means ± SD. The statistical significance of differences between two experimental groups was assessed using *t*‐tests. For multiple comparisons, statistical significance was analyzed by performing one‐way ANOVA followed by Dunnett's test or Tukey's test. *P* < 0.05 was considered statistically significant. Statistical analyses were performed using ezr software 19 (Saitama Medical Center, Jichi Medical University, Saitama, Japan), a graphical user interface for r (The R Foundation for Statistical Computing, Vienna, Austria).

## Results

### Brefeldin A treatment triggers oxidation of TMX1

**Figure 1:**
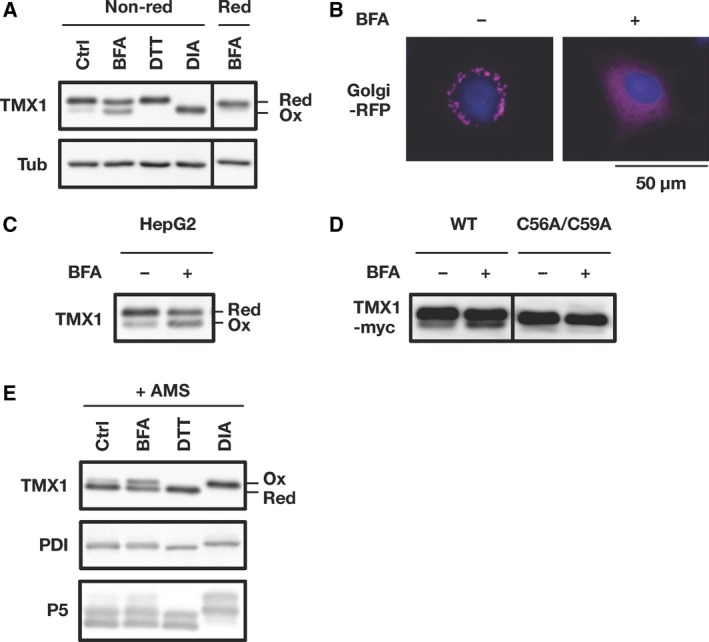
Brefeldin A induces TMX1 oxidation. (A) A549 cells were treated with BFA (0.1 μg·mL^−1^, 6 h), DTT (5 mm, 10 min), or diamide (DIA; 1 mm, 10 min). Cell lysates were separated by performing SDS/PAGE under nonreducing (Non‐red) or reducing (Red) conditions, and the redox status of TMX1 was determined by immunoblotting. Reduced and oxidized forms of TMX1 are indicated. DTT‐ and diamide‐pretreated samples served as the reference for the reduced and oxidized proteins, respectively; α‐tubulin (Tub) was used as a loading control. (B) A549 cells transiently transfected with a construct encoding Golgi‐targeted red fluorescent protein (Golgi‐RFP) were treated with vehicle (−) or 0.1 μg·mL^−1^ BFA (+) for 6 h. Representative fluorescence images of RFP (magenta) are shown. Nuclei were stained with Hoechst 33342 (blue). (C) HepG2 cells were treated with vehicle or 0.1 μg·mL^−1^ BFA for 6 h, and cell lysates were immunoblotted to assess the redox status of TMX1. (D) A549 cells were transiently transfected with myc‐tagged wild‐type TMX1 (WT) or C56A/C59A mutant, and the redox state of exogenous TMX1 protein was analyzed using anti‐myc antibody. (E) A549 cells were treated with BFA (0.1 μg·mL^−1^, 6 h), DTT (5 mm, 10 min), or diamide (DIA; 1 mm, 10 min). After blocking free thiols with NEM, pre‐existing disulfides were reduced with TCEP and this was followed by a second alkylation with AMS. Samples were immunoblotted with antibodies against TMX1, PDI, or P5 to determine the redox status of these proteins. Representative images are shown.

Human lung adenocarcinoma cell line A549 cells were treated with brefeldin A (BFA), a fungal metabolite that inhibits ER‐to‐Golgi transport and thereby causes ER stress 20. The redox state of TMX1 was monitored before and after 6‐h treatment with BFA. To prevent thiol/disulfide exchange during sample preparation, cells were treated with the thiol‐specific alkylating agent *N*‐ethylmaleimide (NEM) before preparing cell lysates. The lysates were separated on SDS/PAGE gels under nonreducing conditions, and the redox state of TMX1 was determined by immunoblotting with an antibody that specifically recognizes the C terminus of TMX1. In this assay, oxidized TMX1, in which a disulfide bond is formed between the active‐site cysteines (C56 and C59), migrates faster because its conformation is more compact than that of reduced TMX1 14. Whereas the majority of TMX1 existed in the reduced form in control A549 cells, in the case of BFA‐treated cells, a band representing the faster‐migrating isoform was detected, and this band corresponded in size to that of oxidized TMX1 in diamide‐treated samples (Fig. 1A). The faster‐migrating band was not detected when the same samples were analyzed under reducing conditions. As expected, Golgi‐targeted red fluorescent protein (RFP) was retained in the ER in BFA‐treated A549 cells and displayed a reticular distribution pattern in the cytoplasm (Fig. 1B). Furthermore, this BFA‐induced conversion of TMX1 to the oxidized form was also observed in human hepatocellular carcinoma cell line HepG2 cells (Fig. 1C). Human TMX1 contains seven cysteine residues. To test whether the mobility shift after BFA treatment reflects disulfide formation between the active‐site cysteines, we examined the redox state of exogenously expressed wild‐type TMX1 and its mutant lacking two cysteines within the active site (C56A/C59A). As shown in Fig. 1D, BFA treatment induced the oxidizing shift of wild‐type TMX1, which was abolished in the cysteine mutant. These results indicated that the TMX1 active site was oxidized after treatment with BFA, and cysteine residues other than C56 and C59 appeared not to contribute to the mobility shift during electrophoresis.

To address the specificity of the BFA effect on TMX1, we investigated the redox status of the two luminal oxidoreductases, protein disulfide isomerase (PDI) and P5, after inducing ER stress by exposing cells to BFA. Here, after blocking free thiols with NEM, pre‐existing disulfides were reduced using tris(2‐carboxyethyl)phosphine hydrochloride (TCEP), after which a second alkylation was performed with 4‐acetamido‐4ʹ‐maleimidylstilbene‐2,2ʹ‐disulfonic acid (AMS). Covalent modification with AMS increases the molecular mass of originally oxidized species by ~0.5 kDa per thiol group, which allows enhanced separation from the reduced form in electrophoresis 17, 18. Assessment of the redox status of TMX1 under these conditions revealed that AMS‐modified proteins were increased in response to BFA and diamide (Fig. 1E), which validated the reliability of this approach for determining protein redox status. BFA treatment did not substantially alter the redox status of either PDI or P5, and the data suggest that BFA specifically affects the redox state of TMX1.

### BFA‐induced TMX1 oxidation precedes upregulation of the ER chaperone immunoglobulin‐binding protein

**Figure 2:**
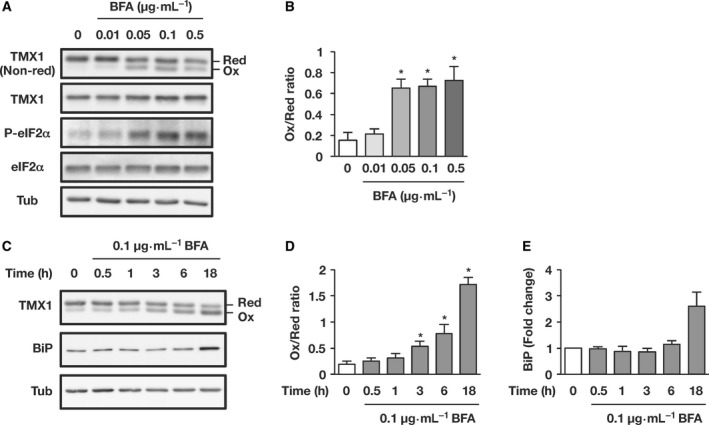
TMX1 oxidizing shift is initiated in the early phase of ER stress response. (A and B) A549 cells were treated with increasing concentrations of BFA for 6 h. Cell lysates were separated by performing SDS/PAGE under nonreducing or reducing conditions, and then analyzed by immunoblotting with the indicated antibodies. Representative images are shown in A. The ratio of oxidized TMX1 to reduced TMX1 (Ox/Red) was quantified using densitometry (B). Data are represented as means ± SD of three independent experiments. Statistical significance was analyzed by performing one‐way ANOVA followed by Dunnett's test for multiple comparisons to the control group. *P < 0.05. (C–E) A549 cells were either left untreated or treated with BFA (0.1 μg·mL^−1^) for the indicated periods, and the shift of the TMX1 redox state was monitored. For detecting the activation of the UPR pathway, blots were probed with anti‐BiP antibody (C). The oxidized/reduced ratio of TMX1 (D) and the fold change of BiP expression normalized to α‐tubulin (E) were quantified using densitometry. The graphs show the means ± SD of three independent experiments. Statistical significance was analyzed by performing one‐way ANOVA followed by Dunnett's test for multiple comparisons to the untreated sample. *P < 0.05.

Brefeldin A treatment induced a dose‐dependent increase in the amount of oxidized TMX1 (Fig. 2A; quantified in Fig. 2B). BFA also elicited a concentration‐dependent upregulation of the phosphorylation of the α‐subunit of eukaryotic translation initiation factor 2 (eIF2α) 21, which confirmed that ER stress was triggered by protein accumulation in BFA‐treated cells. Furthermore, BFA induced a time‐dependent shift of TMX1 to the oxidized form (Fig. 2C; quantified in Fig. 2D). Conversion of TMX1 to the oxidized state was observed within 3 h, which preceded the induction of BiP, a downstream marker of UPR activation 22 (Fig. 2C; quantified in Fig. 2E).

**Figure 3:**
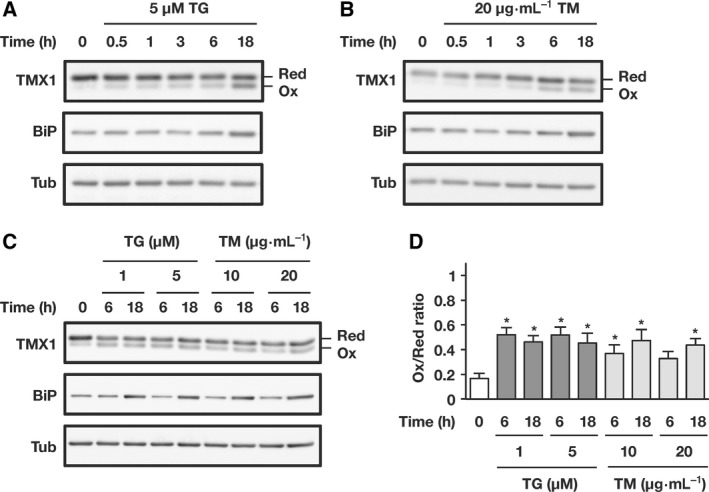
Tunicamycin and thapsigargin only weakly affect the redox status of TMX1. (A and B) A549 cells were treated with 5 μm thapsigargin (TG) or 20 μg·mL^−1^ tunicamycin (TM) for the indicated periods, and the shift of the TMX1 redox state was monitored. For detecting the activation of the UPR pathway, blots were probed with anti‐BiP antibody. The loading control was α‐tubulin (Tub). (C and D) A549 cells were treated with thapsigargin (TG; 1 or 5 μm) or tunicamycin (TM; 10 or 20 μg·mL^−1^) for the indicated periods, and the redox status of TMX1 was determined (C), and the oxidized/reduced ratio of TMX1 was quantified using densitometry (D). Data are represented as means ± SD of three independent experiments. Statistical significance was analyzed by performing one‐way ANOVA followed by Dunnett's test for multiple comparisons to the untreated sample. *P < 0.05.

Next, we tested whether the redox state of TMX1 can be modulated by ER stress inducers other than BFA. A549 cells were treated with either tunicamycin, a potent inhibitor of protein glycosylation, or thapsigargin, which depletes ER calcium stores and thereby causes ER stress. Both agents activated the UPR pathway, which resulted in detectable BiP upregulation at 18 h after treatment (Fig. 3A–C). Although tunicamycin and thapsigargin induced partial oxidation of TMX1 in a time‐dependent manner (Fig. 3A,B), a considerable amount of TMX1 was retained in the reduced form even after overnight exposure to these agents (Fig. 3C; quantified in Fig. 3D). Thus, as compared with BFA, tunicamycin and thapsigargin only weakly affected the redox status of TMX1.

### Reactive oxygen species production does not contribute substantially to BFA‐induced TMX1 oxidation

**Figure 4:**
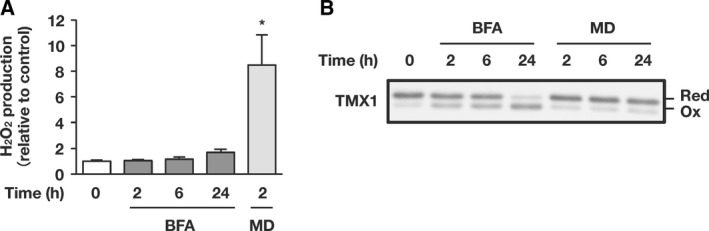
Effect of reactive oxygen species production on TMX1 redox state. (A) A549 cells were treated with 0.1 μg·mL^−1^ BFA for the indicated periods, and the H_2_O_2_ level was measured. Cells treated with menadione (MD; 25 μm) for 2 h were used as the positive control in the experiment. Values shown are relative to the control sample. Data are represented as means ± SD of two independent experiments. Statistical significance was analyzed by performing one‐way ANOVA followed by Dunnett's test for multiple comparisons to the control. *P < 0.05. (B) A549 cells were treated with menadione (MD) or BFA for the indicated periods. The redox status of TMX1 was determined by immunoblotting.

To identify the factor(s) responsible for TMX1 oxidation during ER stress, we first tested the potential involvement of ROS. Measurement of hydrogen peroxide (H_2_O_2_) in vehicle‐ and BFA‐treated cells (Fig. 4A) revealed that the H_2_O_2_ level in cell cultures was not increased until 6 h after BFA treatment, a time by which TMX1 was already oxidized. Longer exposure to BFA (24 h) appeared to marginally affect H_2_O_2_ production: The luminescent signal indicating H_2_O_2_ production showed a slight increase, but this was not statistically significant. By contrast, treatment with menadione/vitamin K3 triggered robust H_2_O_2_ production in a shorter period (2 h) as compared with BFA (Fig. 4A), but did not substantially affect the redox state of TMX1 (Fig. 4B). Thus, TMX1 oxidation induced by BFA does not appear to involve direct oxidative modification by ROS.

### Glutathione is required for maintaining TMX1 in the reduced state

**Figure 5:**
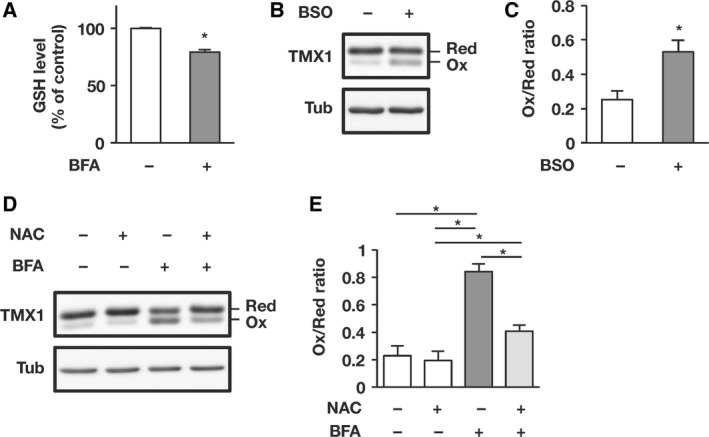
Cellular glutathione consumption induces TMX1 oxidation under conditions of ER stress. (A) GSH levels were measured in cells treated with vehicle or BFA (0.1 μg·mL^−1^) for 6 h. Values relative to the control sample (set as 100%) are shown. Data are represented as means ± SD of three independent experiments. *P < 0.05 (Student's *t*‐test). (B and C) A549 cells were treated with vehicle or BSO (0.5 mm) for 18 h. The redox status of TMX1 was determined by immunoblotting (B). The oxidized/reduced ratio of TMX1 was quantified using densitometry (C). Data are represented as means ± SD of three independent experiments. *P < 0.05 (Student's *t*‐test). (D and E) A549 cells were first incubated without or with 1 mm NAC for 30 min and then treated with vehicle or BFA (0.1 μg·mL^−1^) for 6 h. The redox status of TMX1 was assessed by immunoblotting (D). The oxidized/reduced ratio of TMX1 was quantified using densitometry (E). Data are represented as means ± SD of three independent experiments. Statistical significance was analyzed by performing one‐way ANOVA followed by Tukey's test for multiple comparisons. *P < 0.05.

Glutathione constitutes a major antioxidant pool that maintains the cellular redox environment. We found that 6‐h BFA treatment induced a significant reduction in cellular GSH levels (Fig. 5A), which led us to examine whether diminished GSH levels affect the redox status of TMX1. Thus, we treated cells with l‐buthionine sulfoximine (BSO), an inhibitor of γ‐glutamylcysteine synthetase, to deplete the cellular GSH pool 23. BSO‐mediated inhibition of GSH synthesis resulted in the partial oxidation of TMX1 (Fig. 5B; quantified in Fig. 5C). Conversely, supplementation of intracellular GSH with *N*‐acetyl‐l‐cysteine (NAC) suppressed the BFA‐induced oxidation of TMX1 (Fig. 5D; quantified in Fig. 5E). These results suggest that GSH plays a crucial role in maintaining TMX1 in a predominantly reduced form.

### Oxidized TMX1 is restored to the reduced state after BFA removal

**Figure 6:**
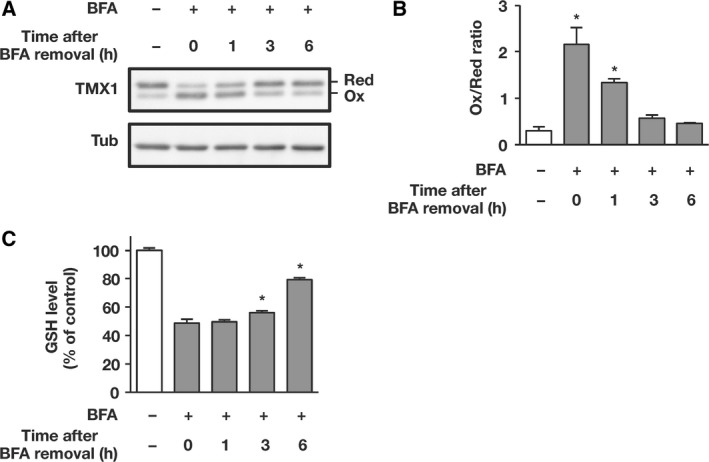
Oxidized TMX1 is restored to the reduced state during recovery from ER stress. (A–C) A549 cells were treated with BFA (0.1 μg·mL^−1^) for 18 h, after which BFA was washed out and the cells were further incubated for the indicated periods before preparing cell lysates. The redox status of TMX1 was determined by immunoblotting (A), and the oxidized/reduced ratio of TMX1 was quantified using densitometry (B). GSH levels were measured and the values relative to the untreated control sample (set as 100%) are shown (C). Data are represented as means ± SD of two independent experiments. Statistical significance was analyzed by performing one‐way ANOVA followed by Dunnett's test for multiple comparisons to the control. *P < 0.05.

To examine the manner in which BFA influences the ER redox status, we incubated BFA‐treated cells in the absence of BFA and monitored the redox state of TMX1 at various time points after BFA removal (Fig. 6A; quantified in Fig. 6B). BFA induced TMX1 oxidation, and the ratio of reduced TMX1 to oxidized TMX1 was reversed relative to control in cells treated with BFA for 18 h (second lane). Recovery of TMX1 from oxidation was initiated within 1 h after washing out BFA, and the relative abundance of the reduced form was increased and returned to the basal level at 6 h after BFA removal. Total GSH levels were restored upon elimination of BFA, suggesting the re‐establishment of the cellular redox homeostasis (Fig. 6C). Thus, TMX1 was reversibly oxidized under conditions of ER stress, and oxidized TMX1 was restored to the reduced state during the recovery process.

## Discussion

**Figure 7:**
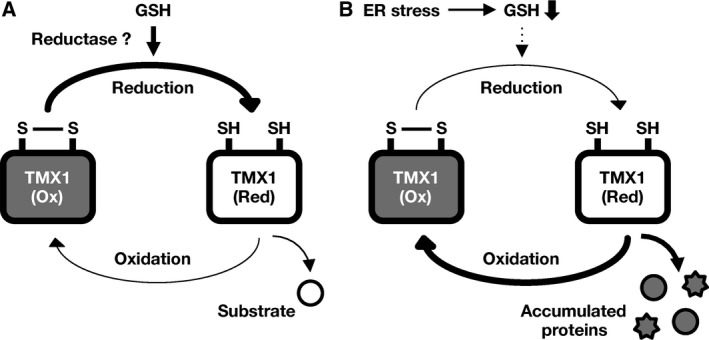
Scheme showing potential mechanisms controlling the redox status and activity of TMX1. (A) TMX1 exists predominantly in the reduced form at steady state. During the process of thiol/disulfide exchange, the active‐site cysteines of TMX1 become oxidized, and they are restored to the reduced state for the next catalytic cycle. Cellular GSH may provide reducing equivalents for maintaining TMX1 in the reduced form. A potential reductase for TMX1 currently remains unidentified. (B) Under conditions of ER stress, protein accumulation triggers oxidation of the TMX1 active‐site cysteines, forming intramolecular disulfides. This induction of TMX1 oxidation does not involve the general protein oxidative damage resulting from reactive oxygen species production. Instead, oxidation of TMX1 may occur as a consequence of thiol/disulfide exchange with substrate proteins accumulated in the ER.

In this study, we investigated the redox changes in the active‐site thiols of the ER oxidoreductase TMX1 in response to ER stress. We demonstrated that BFA‐induced protein accumulation in the ER led to oxidation of the TMX1 active‐site cysteines, which formed intramolecular disulfide bonds. Furthermore, BFA‐induced oxidation of TMX1 was a reversible process, and oxidized TMX1 was restored to the reduced state after removal of the drug. Our findings provide evidence for the existence of a redox‐sensing mechanism that controls the redox properties of ER oxidoreductases (Fig. 7).

TMX1 is predominantly present in the reduced form at steady state, and the enzyme is expected to potentially function as a reductase 14. We examined the changes in the TMX1 redox status under conditions of ER stress and found that BFA‐induced protein accumulation in the ER triggered the oxidation of TMX1 (Figs 1 and 2). ROS generation is widely accepted as a major contributing factor to the oxidative modification of biomolecules, and thus, ROS could represent a candidate TMX1 oxidant. However, our results revealed that after BFA treatment, ROS production was not markedly increased at the point at which a substantial amount of TMX1 had already been oxidized (Fig. 4). Moreover, the redox status of TMX1 was not altered after cells were treated with menadione, a potent ROS inducer. Therefore, BFA‐induced TMX1 oxidation does not appear to occur due to the nonspecific oxidative damage of proteins caused by ROS.

Although the underlying mechanism responsible for TMX1 oxidation currently remains unidentified, we speculate that TMX1 oxidation might occur as a consequence of thiol/disulfide exchange with proteins accumulated in the ER. These proteins could represent potential target substrates of TMX1 and other ER oxidoreductases, and the oxidation of these enzymes would be facilitated at the completion of the substrate reduction in the stressed ER. Notably, TMX1 was reported to preferentially bind to incompletely folded membrane proteins produced under conditions of ER stress 14, which suggests that the catalyzing resolution of disulfide bonds plays a role in coping with protein overload.

Tunicamycin and thapsigargin inhibit protein maturation, which leads to an accumulation of incorrectly folded proteins in the ER 24. Conversely, BFA inhibits the export of proteins (even of proteins that have adopted their native conformation) 25, and this potentially results in an increase in the total amount of protein accumulated in the ER. TMX1 was oxidized most efficiently in response to BFA (Figs 2 and 3), which might be due to the higher rate of protein accumulation in response to BFA than in response to the other ER stress inducers. Intriguingly, BFA has been shown to exhibit properties distinct from those of thapsigargin and tunicamycin in the activation of UPR target genes, which raises the possibility that both the folding status of accumulated proteins and the amount of burden can affect the stress‐sensing mechanism and the downstream ER stress response 26.

The enzymatic activity of TMX1 depends on the active‐site cysteines 12, 13, which can undergo oxidative modification and form intramolecular disulfide bonds. For an oxidoreductase to catalyze continuous rounds of thiol/disulfide exchange, the active redox state of the enzyme must be recovered after each cycle of the reaction 11. BFA reversibly inhibits membrane trafficking, and the Golgi complex reassembles upon BFA removal 27. We found that during recovery after BFA removal, TMX1 returned to the basal reduced state (Fig. 6). The result suggests that cells are equipped with a reductive pathway for adaptation to oxidative modification of proteins. Although several pathways for protein oxidation or disulfide formation have been described 3, the regulatory systems that catalyze protein reduction in the mammalian ER have thus far remained poorly characterized.

With regard to the cellular component responsible for the aforementioned reductive pathway, GSH has been proposed to provide reducing equivalents for the reduction of disulfides 18, 28, 29. In principle, perturbation of oxidative protein folding causes excessive GSH consumption, and the lowered GSH levels would diminish the antioxidant capacity necessary for restoring ER proteostasis 30, 31. In agreement with this notion, BFA treatment lowered the cellular GSH levels in parallel with the oxidizing shift of TMX1, and BSO‐triggered forced depletion of cellular GSH induced the oxidation of TMX1 (Fig. 5). Conversely, replenishment of intracellular GSH levels by using NAC attenuated BFA‐induced TMX1 oxidation. GSH was previously shown to catalyze the reduction of another ER oxidoreductase, ERp57, during the process of recovery from the oxidized state 18. Our current study together with the previous observation emphasizes the requirement of GSH for maintaining the ER redox homeostasis. As the next step in this work, we will determine whether cellular GSH is sufficient for maintaining TMX1 in the reduced state, or whether an alternative enzyme‐catalyzed pathway is required as in the case of the thioredoxin system in the cytosol 32. It will also be intriguing to investigate the crosstalk between TMX1 and PDI family members, which have recently been shown to constitute a hierarchical network in redox cascades 33.

In conclusion, we have demonstrated that protein overload disturbs the ER redox balance and thereby allows the oxidation of TMX1. Our results also indicate that cells harbor a reductive pathway for restoring the redox homeostasis during poststress recovery.

## Author contributions

YM and KH conceived and designed the project. YM performed the experiments. YM and KH analyzed and interpreted the data. YM wrote the manuscript.

## References

[1] Ellgaard L and Helenius A (2003) Quality control in the endoplasmic reticulum. Nat Rev Mol Cell Biol 4, 181–191. 12612637 10.1038/nrm1052

[2] Helenius A , Marquardt T and Braakman I (1992) The endoplasmic reticulum as a protein‐folding compartment. Trends Cell Biol 2, 227–231. 14731479 10.1016/0962-8924(92)90309-b

[3] Oka OB and Bulleid NJ (2013) Forming disulfides in the endoplasmic reticulum. Biochim Biophys Acta 1833, 2425–2429. 23434683 10.1016/j.bbamcr.2013.02.007

[4] Mori K (2009) Signalling pathways in the unfolded protein response: development from yeast to mammals. J Biochem 146, 743–750. 19861400 10.1093/jb/mvp166

[5] Ron D and Walter P (2007) Signal integration in the endoplasmic reticulum unfolded protein response. Nat Rev Mol Cell Biol 8, 519–529. 17565364 10.1038/nrm2199

[6] Kaufman RJ (2002) Orchestrating the unfolded protein response in health and disease. J Clin Invest 110, 1389–1398. 12438434 10.1172/JCI16886 PMC151822

[7] Sevier CS and Kaiser CA (2006) Conservation and diversity of the cellular disulfide bond formation pathways. Antioxid Redox Signal 8, 797–811. 16771671 10.1089/ars.2006.8.797

[8] Ushioda R , Hoseki J , Araki K , Jansen G , Thomas DY and Nagata K (2008) ERdj5 is required as a disulfide reductase for degradation of misfolded proteins in the ER. Science 321, 569–572. 18653895 10.1126/science.1159293

[9] Oka OB , Pringle MA , Schopp IM , Braakman I and Bulleid NJ (2013) ERdj5 is the ER reductase that catalyzes the removal of non‐native disulfides and correct folding of the LDL receptor. Mol Cell 50, 793–804. 23769672 10.1016/j.molcel.2013.05.014 PMC3906653

[10] Ellgaard L and Ruddock LW (2005) The human protein disulphide isomerase family: substrate interactions and functional properties. EMBO Rep 6, 28–32. 15643448 10.1038/sj.embor.7400311 PMC1299221

[11] Hudson DA , Gannon SA and Thorpe C (2015) Oxidative protein folding: from thiol‐disulfide exchange reactions to the redox poise of the endoplasmic reticulum. Free Radic Biol Med 80, 171–182. 25091901 10.1016/j.freeradbiomed.2014.07.037 PMC4312752

[12] Matsuo Y , Akiyama N , Nakamura H , Yodoi J , Noda M and Kizaka‐Kondoh S (2001) Identification of a novel thioredoxin‐related transmembrane protein. J Biol Chem 276, 10032–10038. 11152479 10.1074/jbc.M011037200

[13] Matsuo Y , Nishinaka Y , Suzuki S , Kojima M , Kizaka‐Kondoh S , Kondo N , Son A , Sakakura‐Nishiyama J , Yamaguchi Y , Masutani H *et al* (2004) TMX, a human transmembrane oxidoreductase of the thioredoxin family: the possible role in disulfide‐linked protein folding in the endoplasmic reticulum. Arch Biochem Biophys 423, 81–87. 14871470 10.1016/j.abb.2003.11.003

[14] Matsuo Y , Masutani H , Son A , Kizaka‐Kondoh S and Yodoi J (2009) Physical and functional interaction of transmembrane thioredoxin‐related protein with major histocompatibility complex class I heavy chain: redox‐based protein quality control and its potential relevance to immune responses. Mol Biol Cell 20, 4552–4562. 19741092 10.1091/mbc.E09-05-0439 PMC2770943

[15] Pisoni GB , Ruddock LW , Bulleid N and Molinari M (2015) Division of labor among oxidoreductases: TMX1 preferentially acts on transmembrane polypeptides. Mol Biol Cell 26, 3390–3400. 26246604 10.1091/mbc.E15-05-0321 PMC4591685

[16] Raturi A , Gutierrez T , Ortiz‐Sandoval C , Ruangkittisakul A , Herrera‐Cruz MS , Rockley JP , Gesson K , Ourdev D , Lou PH , Lucchinetti E *et al* (2016) TMX1 determines cancer cell metabolism as a thiol‐based modulator of ER‐mitochondria Ca2+ flux. J Cell Biol 214, 433–444. 27502484 10.1083/jcb.201512077 PMC4987292

[17] Appenzeller‐Herzog C and Ellgaard L (2008) In vivo reduction‐oxidation state of protein disulfide isomerase: the two active sites independently occur in the reduced and oxidized forms. Antioxid Redox Signal 10, 55–64. 17939758 10.1089/ars.2007.1837

[18] Jessop CE and Bulleid NJ (2004) Glutathione directly reduces an oxidoreductase in the endoplasmic reticulum of mammalian cells. J Biol Chem 279, 55341–55347. 15507438 10.1074/jbc.M411409200

[19] Kanda Y (2013) Investigation of the freely available easy‐to‐use software ‘EZR’ for medical statistics. Bone Marrow Transplant 48, 452–458. 23208313 10.1038/bmt.2012.244 PMC3590441

[20] Klausner RD , Donaldson JG and Lippincott‐Schwartz J (1992) Brefeldin A: insights into the control of membrane traffic and organelle structure. J Cell Biol 116, 1071–1080. 1740466 10.1083/jcb.116.5.1071 PMC2289364

[21] Oslowski CM and Urano F (2011) Measuring ER stress and the unfolded protein response using mammalian tissue culture system. Methods Enzymol 490, 71–92. 21266244 10.1016/B978-0-12-385114-7.00004-0 PMC3701721

[22] Kozutsumi Y , Segal M , Normington K , Gething MJ and Sambrook J (1988) The presence of malfolded proteins in the endoplasmic reticulum signals the induction of glucose‐regulated proteins. Nature 332, 462–464. 3352747 10.1038/332462a0

[23] Griffith OW (1982) Mechanism of action, metabolism, and toxicity of buthionine sulfoximine and its higher homologs, potent inhibitors of glutathione synthesis. J Biol Chem 257, 13704–13712. 6128339

[24] Kaufman RJ (1999) Stress signaling from the lumen of the endoplasmic reticulum: coordination of gene transcriptional and translational controls. Genes Dev 13, 1211–1233. 10346810 10.1101/gad.13.10.1211

[25] Preston AM , Gurisik E , Bartley C , Laybutt DR and Biden TJ (2009) Reduced endoplasmic reticulum (ER)‐to‐Golgi protein trafficking contributes to ER stress in lipotoxic mouse beta cells by promoting protein overload. Diabetologia 52, 2369–2373. 19727664 10.1007/s00125-009-1506-5

[26] Shinjo S , Mizotani Y , Tashiro E and Imoto M (2013) Comparative analysis of the expression patterns of UPR‐target genes caused by UPR‐inducing compounds. Biosci Biotechnol Biochem 77, 729–735. 23563539 10.1271/bbb.120812

[27] Fujiwara T , Oda K and Ikehara Y (1989) Dynamic distribution of the Golgi marker thiamine pyrophosphatase is modulated by brefeldin A in rat hepatoma cells. Cell Struct Funct 14, 605–616. 2559814 10.1247/csf.14.605

[28] Cuozzo JW and Kaiser CA (1999) Competition between glutathione and protein thiols for disulphide‐bond formation. Nat Cell Biol 1, 130–135. 10559898 10.1038/11047

[29] Chakravarthi S , Jessop CE and Bulleid NJ (2006) The role of glutathione in disulphide bond formation and endoplasmic‐reticulum‐generated oxidative stress. EMBO Rep 7, 271–275. 16607396 10.1038/sj.embor.7400645 PMC1456887

[30] Chakravarthi S and Bulleid NJ (2004) Glutathione is required to regulate the formation of native disulfide bonds within proteins entering the secretory pathway. J Biol Chem 279, 39872–39879. 15254031 10.1074/jbc.M406912200

[31] Molteni SN , Fassio A , Ciriolo MR , Filomeni G , Pasqualetto E , Fagioli C and Sitia R (2004) Glutathione limits Ero1‐dependent oxidation in the endoplasmic reticulum. J Biol Chem 279, 32667–32673. 15161913 10.1074/jbc.M404992200

[32] Nakamura H , Nakamura K and Yodoi J (1997) Redox regulation of cellular activation. Annu Rev Immunol 15, 351–369. 9143692 10.1146/annurev.immunol.15.1.351

[33] Oka OB , Yeoh HY and Bulleid NJ (2015) Thiol‐disulfide exchange between the PDI family of oxidoreductases negates the requirement for an oxidase or reductase for each enzyme. Biochem J 469, 279–288. 25989104 10.1042/BJ20141423 PMC4613490

